# Effect of CaO in Alkali-Activated Fly Ash Mortar Under Different Curing Temperatures

**DOI:** 10.3390/ma18184250

**Published:** 2025-09-10

**Authors:** David Murillo-Silo, Enrique Fernández-Ledesma, José Ramón Jiménez, José María Fernández-Rodríguez, David Suescum-Morales

**Affiliations:** 1 Área de Explotación de Minas, Universidad de Córdoba, E.P.S de Belmez, Avenida de la Universidad s/n, Belmez, E-14240 Córdoba, Spain; p12musid@uco.es; 2 Área de Ingeniería de la Construcción, Universidad de Córdoba, E.P.S de Belmez, Avenida de la Universidad s/n, Belmez, E-14240 Córdoba, Spain; efledesma@uco.es (E.F.-L.); p02sumod@uco.es (D.S.-M.); 3 Área de Química Inorgánica, Universidad de Córdoba, E.P.S de Belmez, Avenida de la Universidad s/n, Belmez, E-14240 Córdoba, Spain

**Keywords:** CaO, fly ash, alkali-activated materials, curing conditions, microstructure

## Abstract

This work investigates the influence of CaO as a partial substitute for fly ash in alkali-activated fly ash mortars (AAFM), aiming to reduce reliance on conventional thermal curing. Mortars containing 0%, 2%, and 4% CaO were prepared and subjected to two curing regimes: thermal curing at 70 °C for 24 h and ambient curing at 21 °C for 24 h. The materials were thoroughly characterised by XRD, XRF, TGA/DTA, SEM, and particle size distribution, while compressive and flexural strength, density, and porosity were evaluated at 7, 14, and 28 days. The results demonstrated that CaO addition improved mechanical performance in both curing environments, particularly at a 4% substitution level, where compressive strength increased by up to 13.8% under thermal curing conditions. These improvements were associated with the formation of C-S-H and C-A-S-H gels, especially margarite, which contributed to accelerated setting and earlier demoulding. Nonetheless, while CaO incorporation improved mechanical performance and allowed earlier demoulding, it could not fully replicate the effects of heat curing at the studied percentages. Ambient-cured mortars exhibited higher porosity and less compact microstructures than thermally cured samples, which displayed denser, layered morphologies. The study confirms that CaO can act as a partial substitute or reducer for conventional curing, but is not sufficient to enable in situ applications without heat treatment. Future research should explore higher CaO contents in combination with set retarders, intermediate curing regimes, or alternative strategies to balance mechanical performance with energy efficiency.

## 1. Introduction

Achieving carbon-neutral targets has become a shared global responsibility and an urgent priority [1,2]. In line with the Paris Agreement, countries aim to limit global temperature rise to below 2 °C, while pursuing efforts to keep it within 1.5 °C [3,4]. To meet this goal, decarbonisation of major sectors—particularly construction—is essential [5]. Ordinary Portland cement (OPC), which accounts for ~7% of global CO_2_ emissions [6,7,8], remains the dominant binder in construction. Therefore, alternatives such as alkali-activated materials (AAMs) have attracted considerable interest. AAMs based on precursors such as fly ash (FA) and blast furnace slag exhibit high durability and can reduce CO_2_ emissions by 55–75% compared to OPC [9,10,11,12].

Coal combustion remains the dominant fuel source globally, accounting for ~36% of energy production [13]. In 2022, the EU produced 55 Mt of hard coal [14]. Recent geopolitical and energy crises have slowed the expected decline in coal use [15], increasing the production of by-products such as FA and bottom ash (BA). While FA is widely employed in construction as a complementary or substitute material for OPC [16], the growing volumes highlight the need for effective valorisation strategies [17].

OPC production accounts for ~7% of global CO_2_ emissions [7], with ~3 billion tons released annually from limestone decarbonation during manufacture [8]. Consequently, both the scientific community and the construction industry are actively pursuing alternatives to OPC.

Although it is common in the literature to consider the beginning of alkali-activated materials (AAM) around 1970 with Davidovits [18], this is not entirely accurate. The first formally documented reference was by J. Whiting in 1895 [19]. Other researchers preceding Davidovits include Kühl (Germany, 1908) [20], A.O. Purdon (Belgium and France, 1940) [21], and Glukhovsky (Kiev, 1950) [22], among others. Geopolymer binders are alkali-activated aluminosilicates that form a Si–O–Al framework, typically requiring elevated temperatures to react [23]. Their production emits ~55–75% less CO_2_ than OPC [12], positioning AAMs as environmentally friendly, low-energy building materials capable of mitigating the cement industry’s environmental footprint [1,24,25,26].

Despite their sustainability, AAMs often require thermal curing to accelerate geopolymerisation [26,27,28,29,30,31]. Elevated temperatures (60–90 °C) enhance reaction kinetics and mechanical strength [32,33,34,35,36], but they hinder in situ application, increase costs, and compromise environmental benefits [34,37]. To mitigate this, FA is often blended with calcium-rich precursors such as GGBFS [29,38,39].

Refs. [26,27,28,29,30,31,32,33,34,35,36,37,38,39] demonstrate that, unlike conventional two-part AAM systems, the newly developed one-part AAMs consist of a powdered mixture of reactive aluminosilicate precursors and solid alkaline activators, requiring only the addition of water [40,41]. Calcium oxide (CaO) has emerged as an additive of interest in these systems. Upon hydration, CaO releases Ca^2+^ ions and heat, promoting the formation of C-S-H and C-A-S-H gels, which accelerate setting and improve strength development [42]. However, no studies have been conducted using CaO (commercial quicklime) in two-part alkali-activated FA materials, or they are very limited (do not compare heat cure vs. room temperature cure) [43]. Using this strategy, avoiding the heating of samples (in an oven) to facilitate the geopolymerisation process is possible, and the exothermic process of CaO hydration can be exploited (Equation (1)). Although CaO has been studied in one-part systems [42], its role in two-part alkali-activated FA mortars remains scarcely explored, particularly regarding its potential to replace or reduce the need for conventional thermal curing [43].

(1) $$CaO+H_{2}O\to {Ca(OH)}_{2}(Heat)↑$$

The CaO dosage was limited to a maximum of 4% of the FA weight because higher proportions caused rapid setting and poor workability under the experimental conditions employed. In fact, preliminary tests with 8% CaO produced mixtures that were unworkable, confirming that higher dosages are impractical under the experimental conditions employed. While chemical retarders could potentially be used to slow down the reaction kinetics, the present study aimed to evaluate the intrinsic effect of CaO on the hydration and strength development of alkali-activated FA mortars without additional admixtures. This dosage range is consistent with previous studies on one-part alkali-activated systems, where CaO contents of 1–5% have been reported to improve mechanical properties while maintaining manageable setting times [42,43,44,45,46]. By selecting this range, we ensured that the mixtures were workable and comparable under both room-temperature and thermal curing conditions.

Although alkali-activated fly ash mortars have been widely studied, the role of CaO as a partial substitute for fly ash to reduce or replace conventional thermal curing has not been thoroughly explored. This study addresses this gap by analysing the effect of partially replacing FA with 2% and 4% CaO in alkali-activated FA mortars (AAFM) cured under thermal (70 °C, 24 h) and ambient (21 °C, 24 h) conditions. The novelty of this research lies in assessing whether the exothermic hydration of CaO can serve as a substitute or reducer of thermal curing. Mechanical (compressive and flexural strength, density, porosity), chemical (XRD, TGA/DTA), and microstructural (SEM/EDS) characterisations were conducted at 7, 14, and 28 days. The findings provide new insights into the feasibility of incorporating CaO as a strategy to enhance AAFM performance and to enable broader in situ applications without the need for costly thermal curing.

## 2. Materials and Methods

### 2.1. Materials

The raw materials used to manufacture AAFM were as follows: (i) Class F coal fly ash (FA, skeletal density 2188 kg/m^3^) obtained from the combustion of coal at the Los Barrios thermal power plant (Cádiz, Spain), provided by EDP Iberian Generation Platform [44]; reactive CaO (brand CALGOV) with a skeletal density of 1986 kg/m^3^; (iii) natural sand (NS-0/4) with a skeletal density of 2600 kg/m^3^; (iv) commercial NaOH (caustic soda, DIRNA) with 99% purity and a density of 2130 kg/m^3^; and (v) Na_2_SiO_3_ solution (Barcelonesa, S.A., Barcelona, Spain) with a density of 1349 kg/m^3^, composed of 28% SiO_2_, 8% Na_2_O, and 64% H_2_O. Additionally, a superplasticiser, Sikaplast-717, was used as a water-reducing admixture, with a density of 1.21 ± 0.03 kg/dm^3^ and a pH of 10 ± 1. Tap water was used for the preparation of the mixes. Skeletal density was measured at atmospheric pressure using a helium gas pycnometer (MICROTRAC BELPycno Ver 1.14 L., Nuremberg, Germany).

### 2.2. Mix Proportions, Preparation Process, and Curing Conditions

The mix design for the AAFM followed an approach similar to those reported in previous studies [24,45]. The mixtures had volumetric binder-to-aggregate proportions of 1:3, a water-to-binder ratio (by mass) of 0.5, and a superplasticiser-to-binder ratio of 0.002. Three types of mixtures were prepared in this study (Table 1): 100% FA (M0), 98% FA + 2% CaO (M2), and 96% FA + 4% CaO (M4). Since the substitution percentages were small and the densities of FA and CaO were similar, the replacements were performed by weight. Substituting more than 4% of FA with CaO resulted in unworkable mixtures due to rapid setting, consistent with observations in previous studies using calcium-rich compounds [43,47]. A summary of the quantities used in each mix is presented in Table 1.

To prevent excessive temperature rise, a solution containing NaOH, Na_2_SiO_3_, water, and a superplasticiser was prepared and allowed to rest for 30 min before being added to the mortar. Fresh mortar was cast into moulds (previously wrapped in plastic film) with dimensions of 40 mm × 40 mm × 160 mm, following the procedure specified in EN 196-1 [48]. The samples were immediately placed under two different curing conditions:

1. Room temperature (RT): Samples were placed directly into a climatic chamber at 21 °C and 65% relative humidity, without additional heating. They were unmoulded after 24 h and cured at room temperature until the designated testing ages (7, 14, and 28 days). Reference samples (M0) were too fresh to be unmoulded after 24 h, whereas M2 and M4 samples could be conveniently unmoulded.
2. Curing temperature (CT): Samples were heated at 70 °C for 24 h, then unmoulded and subsequently cured at room temperature until the testing ages (7, 14, and 28 days).

#### 2.2.1. XRD and XRF Analysis

The raw materials and hardened samples after 7 and 28 d of curing under both environments were analysed by XRD using a Bruker D8 Discover A25 instrument (Billerica, MA, USA) with Cu Kα radiation (λ = 1.54050 Å, 40 kV, 30 mA). Diffraction patterns were obtained with a goniometric scan from 10° to 70° (2θ) at the speed of 0.016 2θ·s^−1^. The diffractogram peaks of the crystalline phases were compared with reference patterns in the JCPDS library [49]. To determine the elemental composition of raw materials in the form of oxides, a wavelength-dispersive XRF was carried out using ZSX Primus (Rigaku, Tokyo, Japan).

#### 2.2.2. Particle Size Distribution

The particle size distributions of FA and CaO were measured using a Mastersizer S analyser (Malvern Instruments, Malvern, UK), with ethanol as the dispersant. Samples were sonicated for 5 min before measurement.

#### 2.2.3. TGA and DTA

TGA and DTA were performed in a Setaram Setys Evolution 16/18 apparatus, using alumina crucibles in airflow and argon environments for the raw materials and hardened samples. The heating rate was 5°∙min^−1^, and the temperature range was approximately 20–1000 °C.

#### 2.2.4. SEM

The morphology and composition of FA and CaO were determined by SEM, EDS, and backscattered electron imaging (JEOL JSM 7800 F, Tokyo, Japan). The samples were then dusted with a carbon tape, and gold sputtering was used to improve the conductivity of the samples. M0 and M4 were subjected to SEM in both curing environments to study their microstructures.

#### 2.2.5. Fresh and Hardened Properties

Fresh density was evaluated using EN 1015-6 [50]. The consistencies of the fresh mixtures were studied following EN 1015-3 [51]. The compressive and flexural strengths of the hardened mortars were evaluated according to EN 1015-11 [52] at 7, 14, and 28 d in both environments. The dry bulk density and accessible porosity of the water were measured according to EN 1015-10 [53].

## 3. Results

### 3.1. XRF

Table 2 shows the oxide composition of the different materials (FA, CaO, and NS) used in this study. The FA contained the following major oxides: SiO_2_, Al_2_O_3_, and Fe_2_O_3_. According to EN 450-1 [54], this corresponds to high-calcium Class F FA, since these three oxides together comprise more than 50% of the FA. The CaO used had a purity of 97.83%, with the remaining oxides present at levels below 0.8%. The most relevant components of NS-0/4 were SiO_2_ (77.07%) and Al_2_O_3_ (13.36%), confirming that the sand used was silica sand. The high content of aluminosilicates, combined with the additional CaO from the aggregates, played a key role in the successful formation of AAM in this study [55].

### 3.2. XRD Analysis

**Figure 1:**
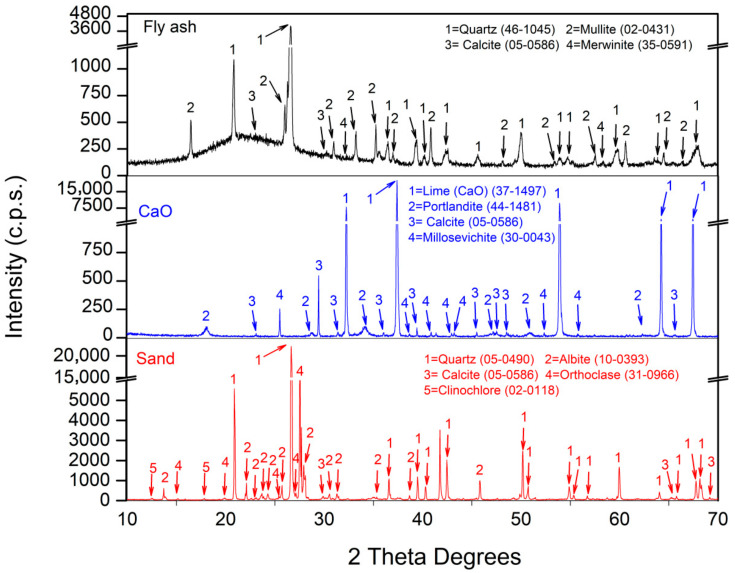
XRD of FA, CaO, and NS-0/4.

According to the XRD analysis of the raw material (Figure 1), the principal crystalline phase of FA was quartz (SiO_2_) (46-1045) [49], while another significant aluminosilicate phase was mullite (2(Al_2_O_3_) SiO_2_) (02-0431) [49]. The calcium-containing crystalline phases include calcite (CaCO_3_) (05-0586) [49] and merwinite (Ca_3_Mg (Si O_2_)_2_) (35-0591) [49]. Similar results have been reported in several FA studies [25,56,57]. As previously mentioned, silica sand was used, with quartz (SiO_2_) (05-0490) [49] as its main crystalline phase. Additionally, minor crystalline phases were observed, such as calcite (CaCO_3_) (05-0586) [49], and aluminosilicates, such as orthoclase (KAlSi_3_O_8_) (31-0966) [49] and albite (NaAlSi_3_O_8_) (10-0393) [49]. In CaO, the dominant peak corresponded to lime (37-1497) [49] with minor peaks of calcium-related minerals including portlandite (Ca(OH)_2_ (44-1481) [49], calcite (CaCO_3_) (05-0586) [49] and millosevichite (Al_2_(SO_4_)_3_) (30-0043) [49].

### 3.3. TGA and DTA

**Figure 2:**
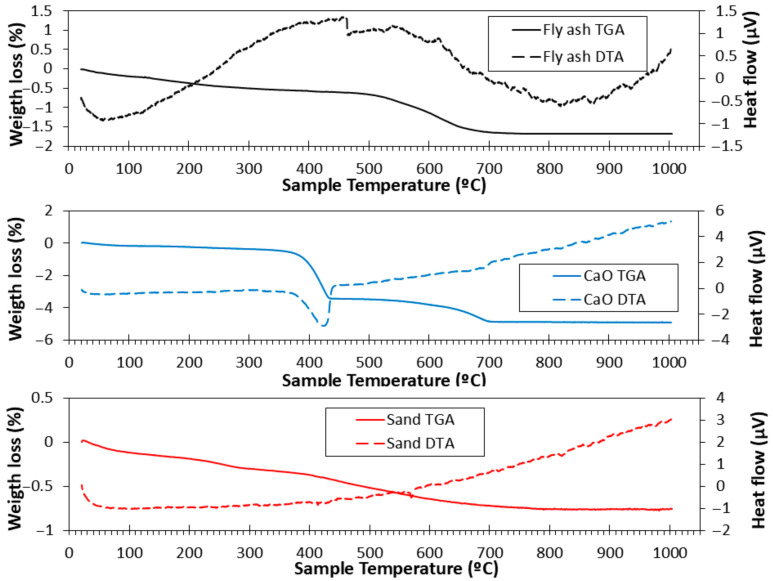
TGA (solid lines) and DTA (dotted lines) curves for FA, CaO, and NS-0/4.

Figure 2 shows the TGA/DTA graphs of FA, CaO, and NS-0/4. For FA, the weight loss gradually increased with temperature. A significant mass loss occurred between 550 °C and 700 °C due to the combustion and oxidation of unburnt coal particles in the FA. Sulphide and iron compounds may also decompose in this temperature range [58]. No appreciable mass loss was observed from 700 °C to 1000 °C, consistent with other studies [56,59]. The DTA curve exhibited an endothermic peak around 460 °C, corresponding to the oxidation and combustion processes detected in the TGA curve [59].

The TGA curve of CaO remained nearly constant from 50 °C to 400 °C, followed by a sharp mass loss between 400 °C and 450 °C due to the dehydroxylation process. The corresponding DTA curve showed a peak in this temperature range, confirming an endothermic reaction [59].

For NS, the TGA curve showed continuous mass loss from 50 °C to 1000 °C. The DTA curve exhibited a peak at 570 °C, corresponding to the α-to-β quartz phase transformation, in agreement with previous reports [25,56,60].

### 3.4. Particle Size Distribution

**Figure 3:**
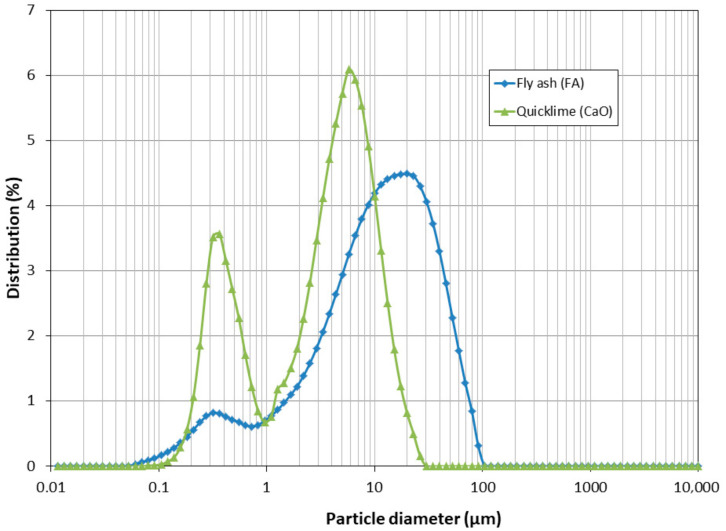
Particle size distribution of FA and CaO.

The particle size distribution of FA (Figure 3) shows a main peak at approximately 19 µm and a smaller peak around 0.30 µm, indicating the presence of a significant fraction of very fine particles (category N) [54]. Similar particle size distributions for FA have been reported in other studies [24,25], which allows comparison and verification that the FA characteristics are consistent across different investigations. For CaO, the particle size distribution exhibited a primary peak at approximately 6 µm (60%) and a secondary peak at around 0.35 µm (35%).

### 3.5. Morphological Characterisation by SEM

**Figure 4:**
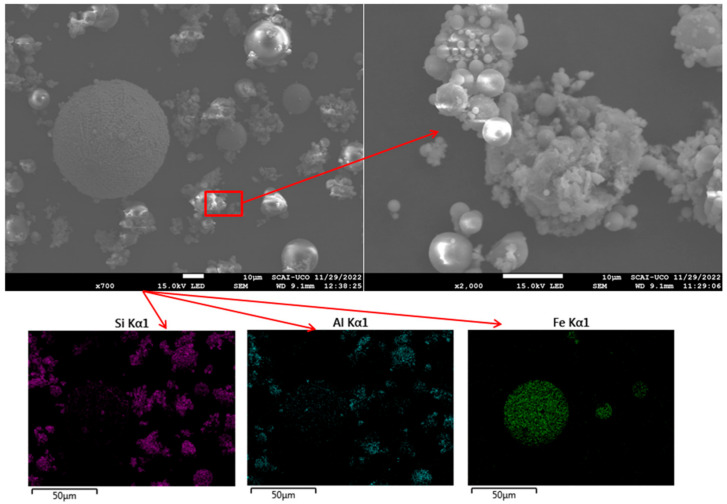
SEM images and EDS mapping of FA.

**Figure 5:**
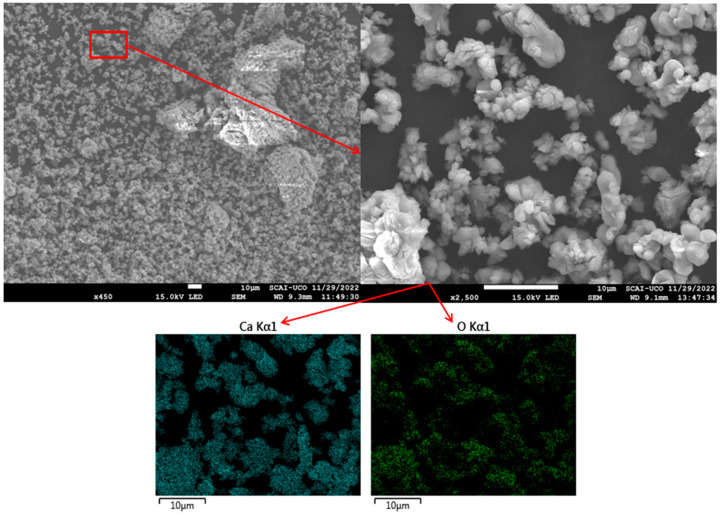
SEM images and EDS mapping of CaO.

SEM micrographs were obtained to characterise the microstructures of FA and CaO. FA particles exhibited a predominantly spherical morphology (Figure 4), with diameters of approximately 20 µm, consistent with the particle size analysis described previously. EDS mapping confirmed that the main elements were Si, Al, and Fe, in agreement with the XRF results (Table 1). Similar SEM observations have been reported in previous studies [34,56]. CaO particles displayed an irregular morphology (Figure 5), with variable dimensions and shapes, as also noted in other research [59]. EDS analysis indicated that the principal elements were Ca and O, corroborating the XRD and XRF data (Figure 1 and Table 1). The higher porosity and presence of cracks in CaO compared to FA may reduce the workability of the resulting mixtures [61].

### 3.6. Bulk Density and Consistency of Fresh Mortar

**Figure 6:**
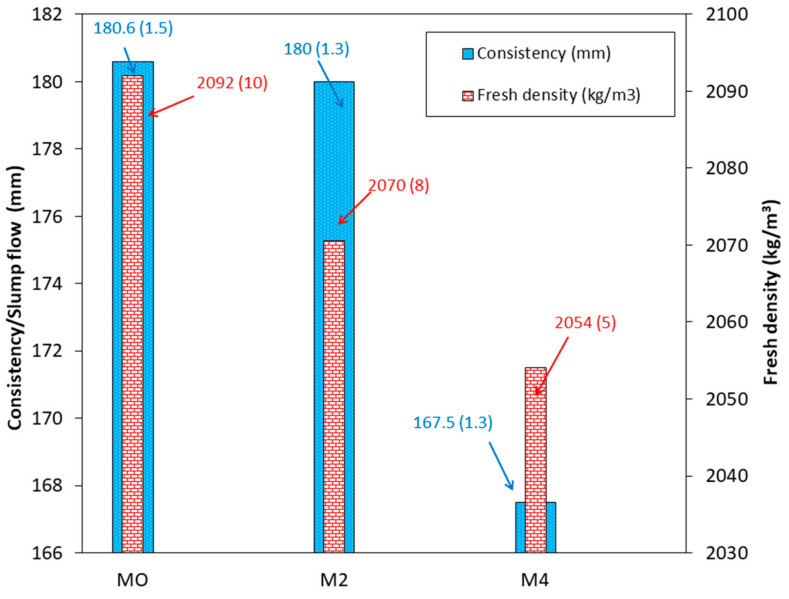
Consistency and fresh density.

Consistency decreased as the CaO content in the binder increased, following the trend M0 > M2 > M4 (Figure 6). This behaviour is attributed to the irregular shape of CaO particles (Figure 5) compared to the spherical morphology of FA (Figure 4), which increases interparticle friction. Although the reduction in consistency is not very pronounced, the mixture containing 4% CaO becomes unworkable after 5 min. This phenomenon, known in the literature as “fast setting” or “accelerated setting” [43], may be caused by the heat of hydration released by CaO. Additionally, free calcium ions from CaO can react with the silica and alumina in FA, leading to the formation of C-S-H and C-A-S-H phases [43]. The fresh density showed a similar trend to consistency, which can be attributed to the higher Ca/Si ratio and is correlated with the reduced workability of the M2 and M4 mixtures (Figure 6) [23,61,62]. It should also be noted that the density of lime is lower than that of FA (2188 kg/m^3^ for FA and 1986 kg/m^3^ for CaO).

### 3.7. Strength Development

**Figure 7:**
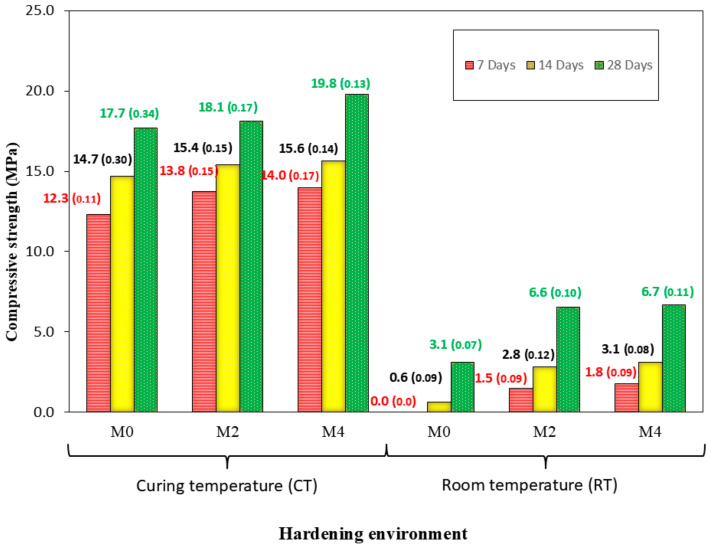
Compressive strength development of AAM with different curing times and curing environments. Mean values are shown, with standard deviation in parentheses.

**Figure 8:**
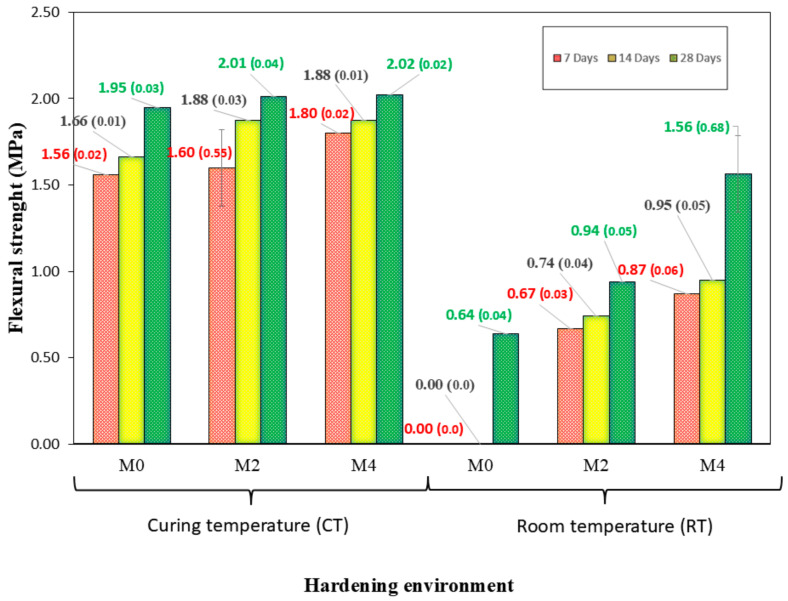
Flexure strength development of AAM with different curing times and curing environments. Mean values are shown, with standard deviation in parentheses.

The mechanical properties of the AAM were evaluated through compressive and flexural tests, with the results shown in Figure 7 and Figure 8. The main finding was that substituting CaO for FA increased compressive strength under both RT and CT curing conditions. This can be attributed to the conversion of CaO to Ca(OH)_2_ upon contact with water, an exothermic process that accelerates the reaction (Equation (1)) [42,63]. Normally, a reduction in precursor content (here, FA) would decrease strength; however, an increase was observed in this study, demonstrating the effectiveness of the proposed strategy of adding CaO to avoid heat curing. Notably, samples containing 4% CaO could be demoulded almost immediately (approximately 30 min)

The best results were obtained with 4% CaO substitution. Under CT conditions, compressive strength increased by 13.8%, 6.1%, and 11.8% at 7, 14, and 28 days, respectively (M0-CT vs. M4-CT). At 7 days under RT, mixes without CaO exhibited negligible strength (0 MPa), while M4-RT reached 1.8 MPa. These results indicate that CaO can act as a partial substitute for FA. Potentially, larger CaO additions could eliminate or reduce the need for heat curing (e.g., from 70 to 40 °C). Nevertheless, simply substituting CaO for thermal curing is not fully effective.

Based on the literature, this behaviour may be explained by several factors: (i) CaO accelerates the alkali-activation process (fast setting) [39,64]; (ii) formation of calcium aluminosilicate hydrate (C-A-S-H) and calcium silicate hydrate (C-S-H) gels to a higher degree [65] owing to the addition of CaO; (iii) heating due to the exothermic process of lime slaking (Equation (1)) [43,66], which enhances the geopolymerisation reactions [25,67]. All these issues will be studied using XRD, TGA/DTA, and SEM.

It is also noteworthy that the strength gain from 2% to 4% CaO was not substantial in either environment, likely due to reduced workability after 5 min. The use of setting retarders might improve workability and allow higher strength. Nevertheless, the results at RT are still relatively low for the CaO percentages studied, indicating that heat curing remains necessary under the conditions investigated.

The trend observed for the flexural strength (Figure 8) is the same as that for the compressive strength. It should be noted that for the mix at RT, if it were not for the use of CaO up to the age of 28 d, the samples did not show resistance. This is indicative of the benefits of using CaO in the mixtures studied: using CaO as a “substitute/assistant” for thermal curing in AAMs. No previous studies used mixtures similar to those used in this study. Again, the difference between 2 and 4% for both environments is not very remarkable.

Again, the results presented do not seem very good. Thermal cure seems inevitable with these dosages, which have also been studied for flexural strength.

### 3.8. Dry Bulk Density and Accessible Porosity

**Figure 9:**
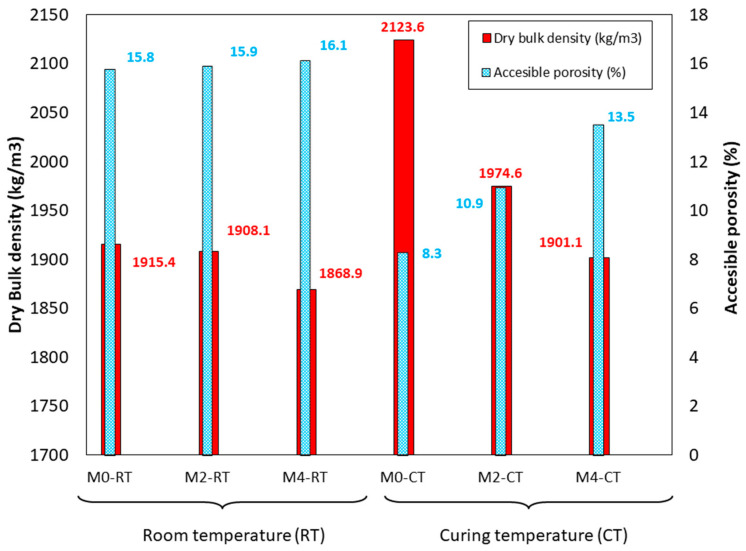
Dry bulk density (kg/m^3^) and accessible porosity (%). (The deviation was less than 0.1 kg/m^3^ for density and less than 0.2% for all the measurements).

Figure 9 presents the dry bulk density (kg/m^3^) and water-accessible porosity (%) of the studied mixtures. No significant differences in dry bulk density were observed between CT and RT samples, likely due to the small percentages of CaO substituted for FA. It is also important to note that the density of FA is higher than that of CaO (2188 kg/m^3^ vs. 1986 kg/m^3^, respectively). Slightly higher dry bulk density values were observed in CT samples compared to RT, indicating that geopolymerisation reactions were more complete under CT conditions. This is further supported by XRD analysis: a reduction in detectable mullite suggests that reactive alumina and silica from FA have participated in the formation of C-A-S-H and N-A-S-H gels, reflecting a more advanced degree of activation, while the persistence of mullite in RT samples indicates incomplete reaction [23,38]. This trend is consistent with the compressive and flexural strength results shown in Figure 7 and Figure 8.

Water-accessible porosity increased with higher CaO content in both curing environments, which may be related to the higher intrinsic porosity of CaO, as observed in the SEM images (Figure 5) [61]. A notable reduction in porosity was observed in CT samples, suggesting that their microstructure was denser than that of RT samples. This observation aligns with the enhanced mechanical properties reported for CT-cured mixtures.

### 3.9. Hydration Products

#### 3.9.1. XRD

**Figure 10:**
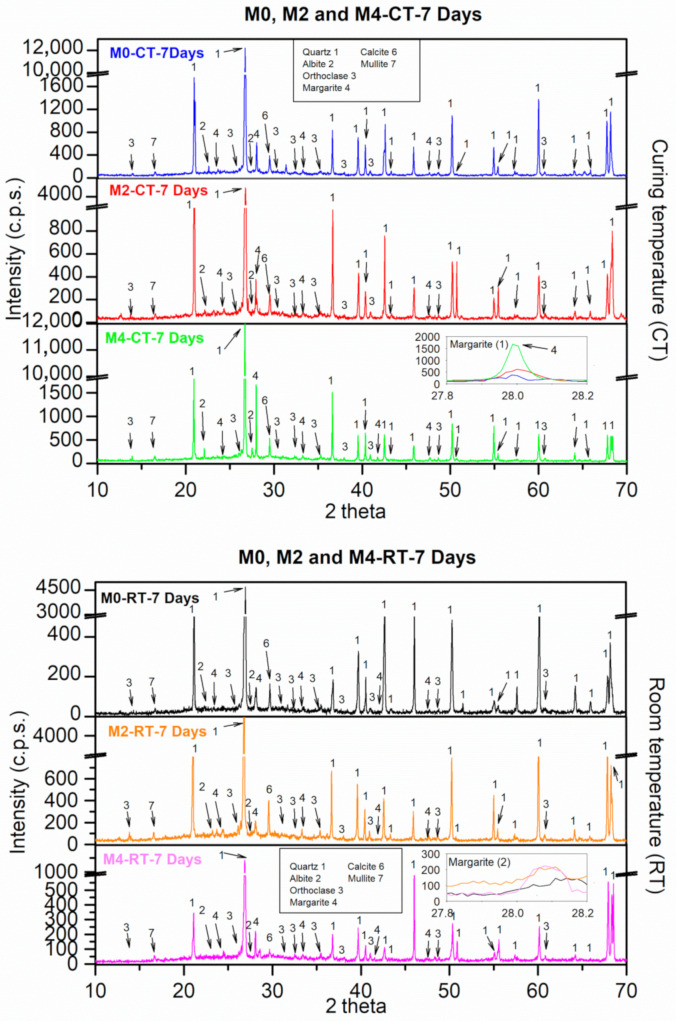
XRD patterns of FA AAM under both curing environments after 7 days of curing.

Figure 10 shows the XRD patterns obtained under the two curing environments at 7 d of age. For the reference sample (M0) under CT, the phases observed were quartz (SiO_2_) (05-0490) [49], albite (NaAlSi_3_O_8_) (10-0393) [49], orthoclase (KAlSi_3_O_8_) (31-0966) [49], and calcite (CaCO_3_) from the sand used (Figure 1). Additionally, mullite (2(Al_2_O_3_)SiO_2_) (02-0431) was found [49]. It should also be noted that quartz and mullite were present in FA. The presence of mullite indicates that the alkaline activation process was not completed by 7 days [67]. Additionally, between 25 and 35° 2ϴ, there was a diffuse band, corresponding to the presence of the vitreous phase (CSH gels) [68]. The main crystalline reaction product is margarite (CaAl_2_(Si_2_)O_10_(OH)_2_) (18-0276) [49]. Similar results were reported by Chi et al. (2013) [69].

Under CT, CaO did not generate new chemical phases. Only slight changes in intensity were observed for the same phases. This may also be due to the small amount of CaO added. In the inset labelled as “Margarite (1)”, we can see that the intensity of this phase was higher when the CaO content was increased. This agrees with the fact that CaO accelerates the alkali activation process (fast setting). Similar results were reported by Pangdaeng et al. [32], who used FA with high calcium content. Hanjitsuwan et al. [64] used calcium carbide residues in mixtures similar to those studied. Chindaprasirt et al. [43] indicated that readily available free calcium ions from calcium hydroxide (CaO + H_2_O) react with the silica and alumina in FA, resulting in C-S-H and C-A-S-H within the matrix. This is in accordance with the obtained mechanical and physical properties (Figure 7, Figure 8 and Figure 9). Notably, no portlandite (Ca(OH)_2_) peaks were detected, despite the addition of CaO. This does not indicate the absence of Ca(OH)_2_; rather, it reflects its rapid consumption in the highly alkaline environment [66]. The freshly formed Ca(OH)_2_ reacts immediately with reactive silica and alumina from the fly ash to form C-A-S-H gel. Therefore, the lack of a portlandite peak provides strong evidence of the effective participation of CaO in accelerating gel formation, particularly at early ages.

The XRD patterns also show a broad amorphous hump between 25 and 40° 2θ, corresponding to the C-A-S-H gel, which is the main binding phase. The XRD patterns of the cured mortars showed a broad amorphous band between 20° and 35° 2θ. This band may partially originate from unreacted fly ash. While some geopolymerisation reactions are expected, the XRD analysis alone does not conclusively quantify the reaction extent, since the amorphous halo may also include contributions from newly formed C-A-S-H and C-S-H gels. Under curing at 70 °C, the apex of the hump shifts slightly to higher angles and becomes narrower, indicating a denser and more polymerised gel network. In contrast, samples cured at room temperature display a broader, lower-intensity hump, suggesting a less connected gel structure. These observations correlate with the higher compressive and flexural strengths observed in thermally cured samples.

At RT, although the phases were the same, a decrease in intensity was observed for the main reaction product, margarite. Moreover, under this environment (RT), a slight broadening of the diffuse band (in this case, ranging from 25 to 40° 2θ) was observed. This indicated a “poorer” alkali-activated reaction under RT, and the reaction improved with temperature [38]. The addition of CaO at RT did not generate new phases. Moreover, in this case, there was no clear increase in the formation of margarite as the addition of CaO increased (see inset labelled “Margarite (2)”). This could be owing to the greater amorphous portion observed at RT than at CT. No similar results were obtained when studying the effects of CaO on the mineralogical compositions (XRD) of similar mixtures at RT. Perhaps, with higher percentages of CaO, these changes would be more noticeable.

**Figure 11:**
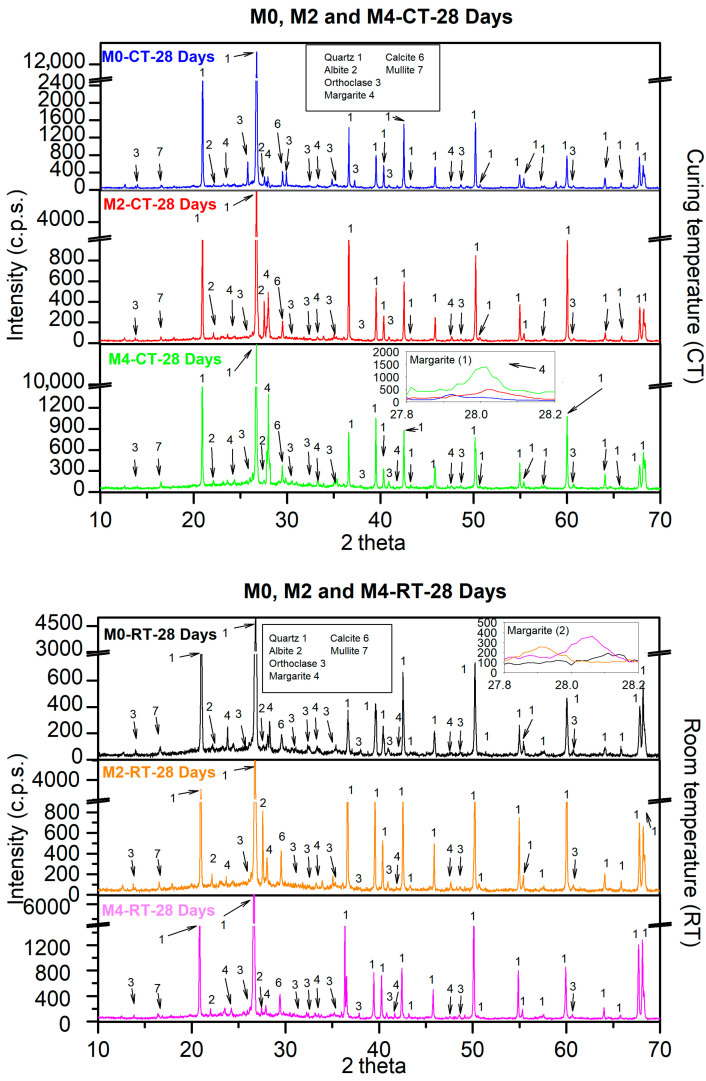
XRD patterns of FA AAM under both curing environments after 28 days of curing.

Figure 11 shows the results for XRD patterns obtained under the two curing environments at an age of 28 days. It should be noted that under CT, the crystallinity seems to be somewhat higher, which could be indicative of a more advanced alkaline activation process than that at 7 d [25,67]. This is in accordance with the fact that the mechanical properties improved over time, as shown in Figure 8 and Figure 9. However, mullite was still present, and reactions continued to occur at the age of 28 d. Again, in the inset labelled as “Margarite (1)”, a higher intensity of this phase is observed as the CaO content increases. For CT, the same observations as for the age of 7 d were made. Additionally, there was no clear increase in the formation of margarite as the addition of CaO increased (see inset labelled “Margarite (2)”).

The patterns also exhibited a broad amorphous hump between 25 and 40° 2θ, characteristic of the C-A-S-H gel network. The apex and width of this hump changed depending on the curing method and CaO content. Samples cured at 70 °C showed a shift of the apex to slightly higher angles and a narrower profile, indicating a denser and more polymerised gel structure. In contrast, room temperature cured samples displayed a broader, lower-intensity hump, suggesting a less connected and less organised gel network. These observations correlate directly with the mechanical performance: the denser gel in thermally cured mortars corresponds to higher compressive and flexural strengths, while the less polymerized structure in room temperature cured mortars explains their comparatively lower strengths.

The addition of CaO influenced the formation of C-A-S-H gel by accelerating the hydration process. As the CaO content increased, the amorphous hump intensity also increased, reflecting enhanced gel formation, which partially compensated for the lower curing temperature in room temperature samples. Overall, the XRD results highlight that the mechanical properties of the AAFM are primarily governed by the evolution and densification of the amorphous gel phase rather than the minor crystalline components.

#### 3.9.2. TGA

**Figure 12:**
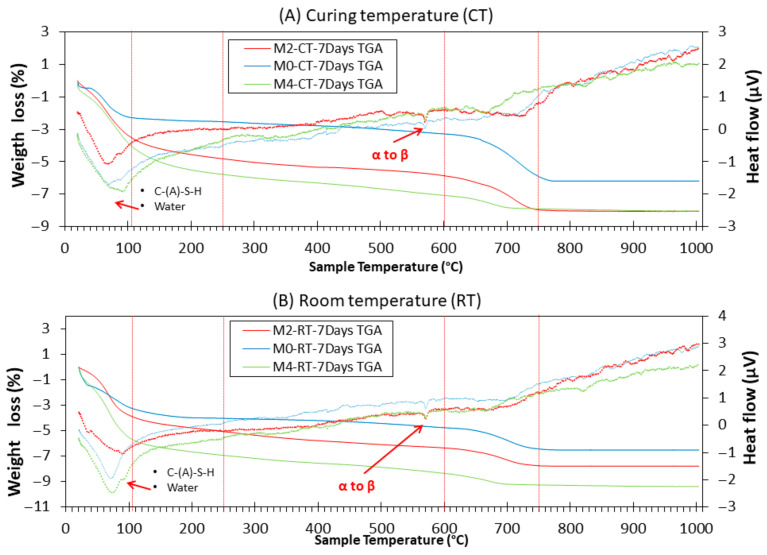
TGA (solid lines) and DTA (dotted lines) of M0, M2, and M4 from both environments at the age of 7 d.

Figure 12 shows the TGA/DTA curves of the M0, M2, and M4 samples after 7 days of CT and RT. Distribution of mass losses corresponding to the composition of hydration products and unhydrated precursors was observed. It should be noted that the comparison of RT and CT samples is made within specific temperature ranges (e.g., <105 °C, 400–500 °C, etc.), which correspond to characteristic decomposition events. Thus, the differences observed reflect the relative evolution of reaction products and bound water content, rather than variations in the initial moisture state of the samples.

As observed in all samples, there is a large mass loss before 250 °C and a tiny mass loss between 600 and 800 °C. The mass loss between 50 and 105 °C was attributed to the loss of physically bound water (peak DTA around 80 °C) [66,70]. Between 105 and 250 °C, the loss of structural water of the matrix and the decomposition of reaction products occurred [24,29,71,72]: C-(A)-S-H and C-S-H. Logically, the mass loss found at RT was higher than that found at CT (because of the moisture loss under CT). Additionally, the mass loss in this range was higher when a higher amount of CaO was added (M4 > M2 > M0). This is in agreement with the XRD insets, which are related to the intensity of margarite (C-A-S-H) [39,64]. C-A-S-H and C-S-H are known gels. The number of gels was directly related to the mechanical strength of the specimens [65]. This explains why the compressive and flexural strengths increased with the addition of CaO (see Figure 7 and Figure 8) at the CT. From 250 to 600 °C, several processes were observed. From approximately 250–400 °C, a continuous weight loss was observed in all samples. This may be owing to the slight presence of unburnt FA (it was not very clear at this magnification in the hardened TGA, but it was observed in the raw FA, as shown in Figure 2) [71]. TGA and DTG showed mass loss associated with dehydration of C-A-S-H gel and other hydrated phases. No distinct portlandite decomposition peak (~450 °C) was observed. This absence is consistent with XRD results and confirms that Ca(OH)_2_, formed by the hydration of CaO, was rapidly consumed in reactions with fly ash’s reactive silica and alumina to produce additional C-A-S-H gel. In DTA, the transition from alpha to beta quartz in NS was also observed at approximately 570 °C [73,74]. Additionally, the peak found around 630 °C in the DTA curves was due to the decomposition of CaCO_3_ in accordance with the XRD results. The greatest weight loss occurs approximately between 600 and 750 °C. In this stage, the loss of CaCO_3_ occurs [24,25,75]. Between 750 °C and 1000 °C, the hydroxyl groups (OH^−^) were lost.

**Figure 13:**
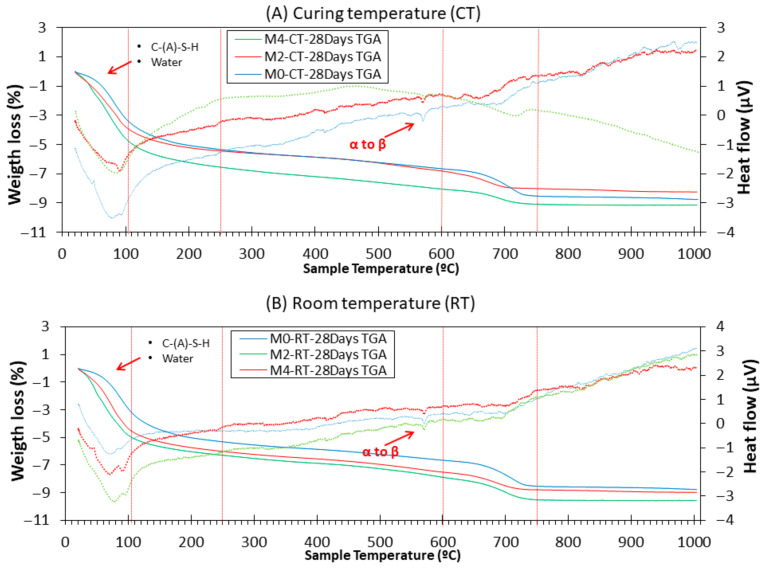
TGA (solid lines) and DTA (dotted lines) of M0, M2, and M4 from both environments at the age of 28 d.

Figure 13 shows the TGA/DTA curves of the M0, M2, and M4 samples after 28 d of CT and RT. In the first stage (up to 250 °C), we found that the weight loss owing to the decomposition of C-A-S-H and C-S-H was higher with a higher CaO content. This observation was valid for CT and RT. After corroborating the XRD and TGA/DTA results showing that the addition of CaO increases C-A-S-H, the effectiveness of this strategy was reiterated. Future studies could aim to find a “trade-off” between temperature decrease (thermal curing) and lime addition or substitution. This can decrease the carbon footprint produced by the thermal curing of conventional alkaline-activated materials.

The quantified mass losses within the characteristic temperature ranges (Table 3) provide direct evidence of the influence of CaO addition and curing conditions on the formation of hydration products, confirming the conclusions drawn from the thermal analysis.

#### 3.9.3. SEM

**Figure 14:**
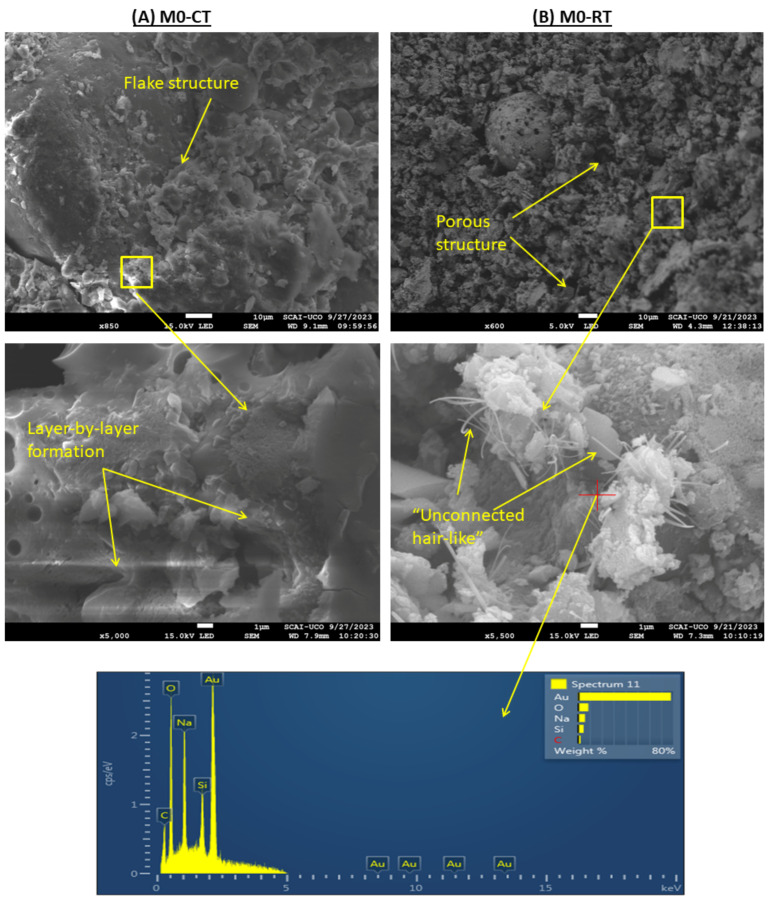
SEM and EDS of M0 under CT (**A**) and M0 under RT (**B**): the presence of Au (from the coating applied for SEM imaging) and C (originating from the alkali-activated material derived from coal fly ash) corresponds to the influence of the research method, not to the intrinsic composition of the sample.

The strength development can be explained by SEM analysis. Figure 14 shows the morphologies of (A) M0-CT and (B) M0-RT after 7 days of curing. At CT (A), the main reaction products exhibited a flake-like structure, primarily formed around the FA particles. This morphology and microstructure are very similar to those observed in conventional heat curing studies [28,31]. Additionally, the overall microstructure appeared dense, and layer-by-layer formation could be observed at higher magnifications, indicating effective geopolymerisation [76]. In contrast, the RT-cured sample (B) showed a highly porous microstructure, which likely contributes to the lower strength of M0-RT compared to M0-CT. At higher magnification, the reaction products at RT appeared as “unconnected hair-like” structures, which some studies attribute to Na_2_SiO_3_ phases [67,77,78,79]. EDS analysis confirmed that these structures were mainly composed of sodium and silicon.

**Figure 15:**
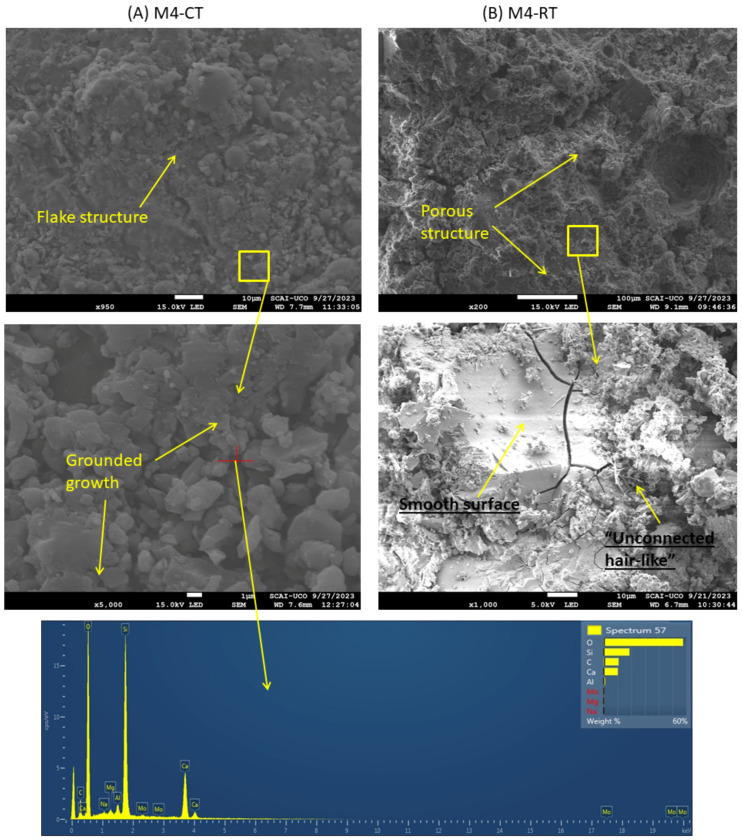
SEM and EDS of M4 under CT (**A**) and M4 under RT (**B**): the detected C originates from the alkali-activated material derived from coal fly ash and corresponds to the influence of the research method, not to the intrinsic composition of the sample.

Figure 15 presents the morphologies of (A) M4-CT and (B) M4-RT after 7 days of curing. At low magnification, the addition of CaO did not induce significant changes at CT. However, at high magnification, the layer-by-layer formation observed in M0-CT (Figure 15A) was less evident. Instead, agglomerates identified by EDS as C-A-S-H were observed [80], which was corroborated by XRD and TGA/DTA analyses. This confirms that the free calcium ions from CaO react with the silica and alumina of FA, producing additional C-S-H and C-A-S-H within the matrix [43]. This corresponds to the increased compressive strength observed for M4-CT compared to M0-CT.

At RT (B), the microstructure remained porous, though slightly denser than that of M0-RT (Figure 14B vs. Figure 15B). Higher magnification revealed smooth surfaces beneath the porous structure, albeit with numerous cracks. The SEM voltage had to be reduced from 15 kV to 5 kV to capture these details properly. This subtle microstructural change may explain the slight strength improvement seen in M4-RT. Overall, the SEM observations are consistent with the XRD and TGA/DTA results, and they align with the mechanical and physical properties reported in Figure 6, Figure 7, Figure 8 and Figure 9.

## 4. Conclusions

In this study, the effect of partially replacing fly ash (FA) with CaO (0, 2, and 4% by weight) was analysed in alkali-activated fly ash mortars under two curing conditions: (i) thermal curing at 70 °C for 24 h (CT) and (ii) ambient curing at 21 °C for 24 h (RT). The novel aspect of this study lies in the use of the exothermic hydration of CaO as a potential substitute or reducer for conventional thermal curing. The main findings are summarised as follows:

1. Substituting FA with CaO improved compressive and flexural strength in both RT and CT environments, with the best results obtained at 4% CaO. Although CT still provided higher strength than RT, CaO addition enhanced mechanical properties even under ambient curing, showing partial effectiveness of the proposed strategy.
2. Despite a reduction in precursor content, CaO substitution led to strength improvements. This indicates that CaO can serve as a partial substitute or reducer of thermal curing, enhancing early-age reactivity and mechanical performance.
3. At the studied levels, increasing CaO content beyond 4% was not feasible due to fast setting and poor workability, which hindered practical application without the use of retarders.
4. XRD and TGA/DTA confirmed that the enhanced performance was associated with the formation of C-S-H and C-A-S-H gels (notably margarite), which accelerated setting and enabled earlier demoulding (≈30 min for 4% CaO).
5. SEM analysis revealed that CT samples exhibited denser, flake- and layer-like microstructures, while RT samples remained more porous and weakly connected. The addition of CaO slightly improved RT microstructures, though not to the level of CT.

Overall, the study demonstrates that the addition of CaO can improve the performance of alkali-activated fly ash mortars and partially reduce reliance on conventional thermal curing. However, under the conditions studied (≤4% substitution), CaO alone cannot fully replicate the effects of heat curing; therefore, is not yet sufficient to enable in situ applications without some form of thermal assistance.

Future research should explore the following: (i) CaO as an additive without reducing FA content, (ii) higher CaO percentages in combination with set retarders to mitigate rapid setting, and (iii) intermediate curing regimes (e.g., 40 °C) to balance mechanical performance with energy efficiency.

## Figures and Tables

**Table 1:** Mix proportion compositions (kg/m^3^).

Mortar Type	FA	CaO	Water Reducing Admixture	Water	Sand 0/4	NaOH	Na_2_SiO_3_
M0	450	-	0.88	879	1316	57	224
M2	441	9	0.88	879	1316	57	224
M4	432	18	0.88	879	1316	57	224

**Table 2:** XRF chemical composition of the raw materials.

Oxides	FA	CaO	Natural Sand (NS-0/4)
Na_2_O	0.55	-	0.81
MgO	0.47	0.48	0.95
Al_2_O_3_	15.77	0.06	13.36
SiO_2_	74.46	0.14	77.07
P_2_O_5_	0.20	0.22	0.13
SO_3_	0.47	0.74	0.11
Cl^−^	0.08	0.05	-
K_2_O	0.97	0.15	3.47
CaO	1.27	97.83	0.71
TiO_2_	1.47	-	0.82
MnO_2_	0.05	-	0.08
Fe_2_O_3_	4.15	0.03	3.98
CuO	-	-	-
ZnO	-	-	-
SrO	0.04	0.27	0.01
ZrO_2_	0.04	-	0.02
BaO	-	-	-
TOTAL	100	100	100

**Table 3:** Total mass loss in thermogravimetric analysis (TGA/DTA) for the different mixes.

Mixes	RT-105 °C	105–250 °C	250–600 °C	600–1000 °C
M0-RT-7 D	3.279	0.741	0.734	1.031
M2-RT-7 D	3.979	1.106	1.267	1.417
M4-RT-7 D	5.711	1.212	1.413	1.549
M0-CT-7 D	2.288	0.910	0.746	1.131
M2-CT-7 D	3.508	1.305	1.289	1.549
M4-CT-7 D	4.032	1.74	1.456	1.608
M0-RT-28 D	3.277	1.284	1.332	1.461
M2-RT-28 D	3.576	1.435	1.499	1.488
M4-RT-28 D	4.576	2.040	1.583	1.598
M0-CT-28 D	3.110	1.445	1.491	1.502
M2-CT-28 D	3.456	1.706	1.654	1.546
M4-CT-28 D	4.126	2.069	1.702	1.604

## Data Availability

The original contributions presented in this study are included in the article. Further inquiries can be directed to the corresponding authors.
